# Elevated Axonal Protein Markers Following Repetitive Blast Exposure in Military Personnel

**DOI:** 10.3389/fnins.2022.853616

**Published:** 2022-04-29

**Authors:** Rany Vorn, Rosanne Naunheim, Chen Lai, Chelsea Wagner, Jessica M. Gill

**Affiliations:** ^1^School of Nursing, Johns Hopkins University, Baltimore, MD, United States; ^2^National Institute of Nursing Research, National Institutes of Health, Bethesda, MD, United States; ^3^Department of Emergency Medicine, Washington University Barnes Jewish Medical Center, St. Louis, MO, United States; ^4^Center for Neuroscience and Regenerative Medicine, Uniformed Services University of Health Sciences, Bethesda, MD, United States; ^5^School of Nursing and Medicine, Johns Hopkins University, Baltimore, MD, United States

**Keywords:** concussion, biomarker, axonal, repetitive blast, low level blast

## Abstract

Blast exposures that occur during training are common in military personnel; however, the biomarkers that relate to these subtle injuries is not well understood. Therefore, the purpose of this study is to identify the acute biomarkers related to blast injury in a cohort of military personnel exposure to blast-related training. Thirty-four military personnel who participated in the training program were included in this study. Blood samples were collected before and after repetitive blast-related training on days 2 (*n* = 19) and days 7 (*n* = 15). Serum concentration (pg/mL) of tau, glial fibrillary acidic protein (GFAP), neurofilament light chain (NfL), and phosphorylated tau181 (p-tau181) were measured using an ultrasensitive immunoassay platform. We observed that serum p-tau181 concentrations were elevated after exposed to repetitive blast on days 2 (z = −2.983, *p* = 0.003) and days 7 (z = −2.158, *p* = 0.031). Serum tau (z = −2.272, *p* = 0.023) and NfL (z = −2.158, *p* = 0.031) levels were significantly elevated after exposure to repetitive blasts on days 7. Our findings indicate that blast exposure affects serum biomarkers indicating axonal injury.

## Introduction

Blast exposures are a prominent feature of injury in military training and combat related due to the use of improvised explosive devices (Ritenour and Baskin, 2008). The impact of blast can be divided into four classification categories: primary, secondary, tertiary, and quaternary based on the mechanism of blast injury such as impact of the overpressure wave with body surfaces, penetrating fragmentation or blunt injury, and/or direct exposure to toxic inhalant, burn, and asphyxia (Wolf et al., 2009). History of blast traumatic brain injuries (bTBI) of all severities can be associated with long-term neurobehavioral sequelae (Lippa et al., 2020) and have been linked to a risk for neurodegenerative processes (McKee et al., 2013; Barnes et al., 2018). The primary bTBIs (mild TBI or concussion) are account for 80% of the bTBI in military population, yet the biological mechanism of blast affects the brain physiological function are limited. Most common concussion or mild TBI occur in athletes, military training, and combat (Lehman et al., 2012). Concussion may have a temporary effect on neurological and cognitive impairment which resolve within day to weeks in most individuals impacted (Frieden et al., 2015; Dikmen et al., 2017). When concussions or bTBIs occur multiple times, individuals are at greater risk for chronic neurological dysfunction and long-term consequences for neurodegeneration (McKee et al., 2013; McKee and Robinson, 2014; McAllister and McCrea, 2017). To date, there is little known about the impact of repetitive blast exposures on fluid biomarkers, and the management of subconcussive impacts is based primarily on clinical symptom presentation. Therefore, a better understanding of temporal changes in proteins that are related to neuronal injuries is important to determine impacts of these exposures, and for future clinical management.

In contrast to low-level blast exposures, blunt TBI blood-based biomarkers have been relatively well investigated, including glial fibrillary acidic protein (GFAP), neurofilament light chain (NfL), tau, and phosphorylated tau (p-tau) (Bogoslovsky et al., 2017; Rubenstein et al., 2017; Wang et al., 2018). Elevated blood concentrations of GFAP, and NfL level have been observed following a TBI and sports concussion, a component of the cytoskeleton of astrocytes, and neuro-axonal damaged (Shahim et al., 2014; Gill et al., 2018; McCrea et al., 2020; Meier et al., 2020; Tschiffely et al., 2020; Giza et al., 2021). Previously, we have observed axonal marker levels of NfL and tau proteins were elevated in military training exposure to moderated blast exposure (≥5 psi) (Edwards et al., 2020). Tau is a microtubule-associated protein that plays a crucial role in regulating microtubule dynamics, axonal transport, and neurite outgrowth, and all these functions of tau are modulated by site-specific phosphorylation (Barbier et al., 2019). In a previous study it was shown that serum tau and p-tau (231) levels were elevated in severe TBI patients and associated with poor outcomes at 6 months (Rubenstein et al., 2017). In combat-related repetitive mild TBI were reported higher exosomal p-tau181 and associated with neuropsychological symptoms (Kenney et al., 2018). Accumulated activity of tau phosphorylation is linked to synaptic impairment, neuronal dysfunction, and the formation of neurofibrillary tangles (NFTs), a key pathological feature of several neurodegenerative diseases and chronic traumatic encephalopathy (CTE) (Rajmohan and Reddy, 2017; Katsumoto et al., 2019). Recently, p-tau181 and 217 are a potential biomarkers for Alzheimer’s Disease (Thijssen et al., 2020, 2021). To our knowledge, the effect of repetitive low-level blast exposure on peripheral biomarkers of axonal damage is not well understood. Specifically, the effect of acute blast exposure on serum p-tau181 level is not known in the clinical cohort. Therefore, the purpose of this study was to investigate the feasibility of acute biomarkers changes after blast-related training exposure in military personnel.

## Materials and Methods

### Study Participants

**FIGURE 1:**
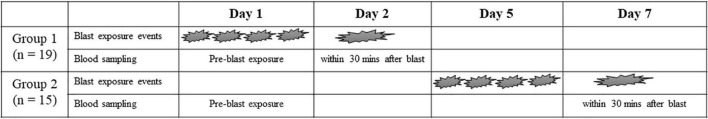
Summary of blast exposure schedule in both group Participants were exposed to four light charges which less than 1 pound (0.03, 0.07, 0.11, 0.15) of net explosive weight (NEW) and a single heavy charges (10.44 pounds of NEW).

This study protocol was reviewed and approved by Institutional Review Board Committee at Washington University and U.S Army Fort Leonard Wood. Written informed consents were received prior to enrolling in this study. All participants were male military personnel (*n* = 34) who participated in the breaching training program at Fort Leonard Wood. The blood samples were collected on day 1 (baseline) and after post-training on day 2 and day 7 of the training program. Participants have experienced the same types of explosive charges, and the light charges were less than 1 pound (0.03, 0.07, 0.11, and 0.15) of net explosive weight (NEW) and the heavy charges were 10.44 pounds of NEW. The heavy charges in this study were similar to 4.35 pounds per square inch (psi) blast overpressure in the previous study (Boutté et al., 2019). The length of the detonation cord varied with the charges. Group 1 participants (*n* = 19) were exposed to 4 light blasts during training on day 1 and exposed to a single heavy blast on day 2. Group 2 participants (*n* = 15) were exposed to 4 light blasts on day 5 and a single heavy blast on day 7 of training. Blood was collected before and after training within 30 min of blast exposure. The representative figure of this study design is presented in Figure 1.

### Protein Quantification

Venipuncture blood was collected, centrifuged (15 min, 1,500 *g*, room temperature), and frozen (−80^°^C) in aliquots within 60 min of sample collection. Samples were shipped on dry ice to the National Institutes of Health for protein quantification. Serum samples were analyzed in duplicate using the Single Molecule Array (SIMOA) Assay (Quanterix, Lexington, MA) for measurement of tau, GFAP, NfL, and p-tau181 concentration on a HD-X Analyzer™. Samples were diluted 4-fold for measurement. Briefly, four distinct, dye-encoded bead populations presented with analyte-specific capture antibodies were first incubated with samples and biotinylated detector antibodies. The target molecule present within each sample was captured by capture beads and labeled with the corresponding detector antibodies. The bead-conjugated immunocomplex was thoroughly washed and labeled with streptavidin-conjugate β-galactosidase. Following a final wash, resorufin β-D-galactopyranoside was added. The bead-conjugated immunocomplexes were loaded on the SIMOA array disc, which is designed to enable imaging of each bead via their encoded dyes and fluorescent substrate generated signals. The number of bead-containing wells producing positive signals was proportional to the number of target molecules within the sample for each plex. The average number of enzymes per bead (AEB) of each sample fit into a four-parameter logistic curve plotted using the known concentration of the calibrators. The correlation was confirmed for the accuracy of fit and for the conversion of AEB values to concentrations. The average coefficient of variation of biomarkers were no higher than 25%; and the lower limits of quantifications (LLOQs) for tau, GFAP, NfL, and p-tau181 were 0.212, 1.868, 0.964, and 1.352 pg/ml, respectively.

### Statistical Analysis

Statistical analyses were conducted using Statistical Package for the Social Science (SPSS) version 28 (Armonk, NY, IBM Corp.). GraphPad Prism version 9.3 was used to generate a graph in this study (GraphPad Software, La Jolla, CA). Wilcoxon signed-rank test was used to assess changes in proteins concentration before and after blast-related training. Statistical significance was considered with *p* < 0.05.

## Results

All participants in this study were male with a mean age of 31.21 years (*SD* = 4.49) and with a range of 24–38 years of age for group 1. The mean age of group 2 was 35.40 years (*SD* = 8.16) with a range of 26–52 years of age. The majority of them were White for both groups (85.5 and 86.7%). Demographic characteristics of the study participants are shown in Table 1.

**TABLE 1:** Sample characteristics of the study participants.

	Group 1 (*N* = 19)	Group 2 (*N* = 15)
Age, year, mean (*SD*)	31.21 (4.49)	35.40 (8.16)
Male, no. (%)	19 (100)	15 (100)
**Race, no. (%)**		
White	17 (85.5)	13 (86.7)
Missing	2 (10.5)	2 (13.3)
**Previous concussion, no (%)**		
0	19 (100.0)	14 (93.3)
5	0 (0.0)	1 (6.7)

**FIGURE 2:**
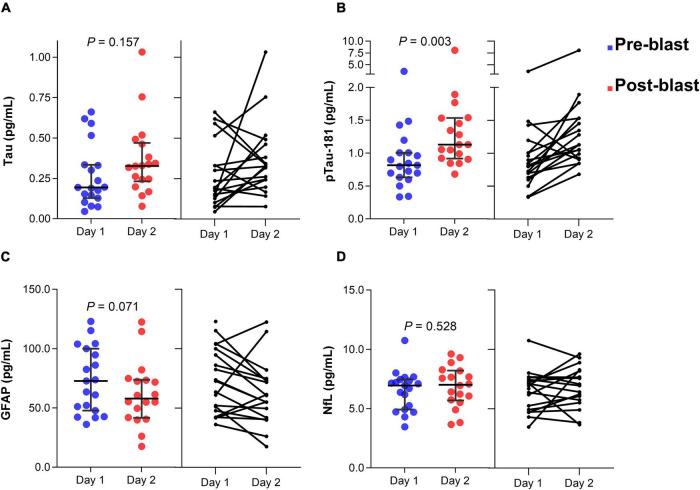
Dot plots of serum tau **(A)**, p-tau181 **(B)**, GFAP **(C)**, and NfL **(D)** concentration before and after blast exposure at day 2. The horizontal line in each box represents the median, with the error bars representing the interquartile range. Wilcoxon signed-rank tests were used to analyze the differences before and after blast exposure. p-tau181, phosphorylated tau181; GFAP, glial fibrillary acidic protein; NfL, neurofilament light chain.

In group 1, serum tau concentration was not significantly different after day 2 blast exposure (z = −1.415, *p* = 0.157) (Figure 2A). The median concentration of tau at day 1 was 0.19 pg/mL (25–75th percentile, 0.12–0.39) and day 2 blast exposure was 0.33 pg/mL (0.23–0.47). Serum p-tau181 concentration was significantly elevated after day 2 blast exposure (z = −2.983, *p* = 0.003) (Figure 2B). The median concentration of p-tau181 at day 1 was 0.81 pg/mL (0.62 − 1.05) and 1.13 pg/mL (0.92-1.53) in day 2. Concentration of GFAP level was trending significant after day 2 blast exposure (z = −1.807, *p* = 0.071) (Figure 2C). The level of NfL was not significantly different after day 2 blast exposure (z = −0.631, *p* = 0.528) (Figure 2D).

**FIGURE 3:**
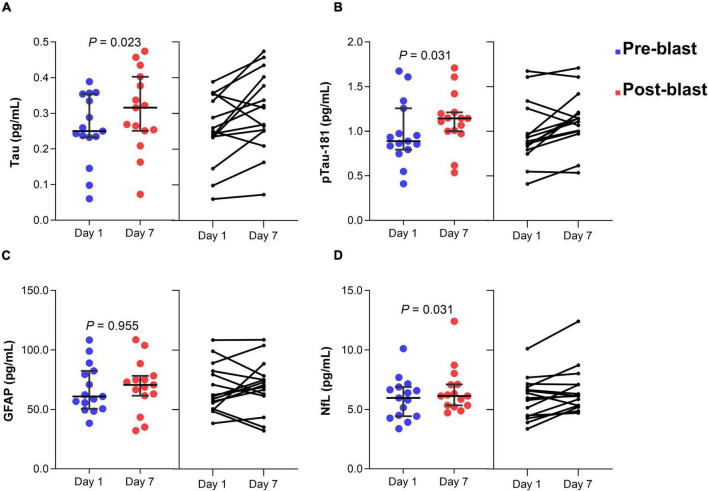
Dot plots of serum tau **(A)**, p-tau181 **(B)**, GFAP **(C)**, and NfL **(D)** concentration before and after blast exposure at day 7. The horizontal line in each box represents the median, with the error bars representing the interquartile range. Wilcoxon signed-rank tests were used to analyze the differences before and after blast exposure p-tau181, phosphorylated tau-181; GFAP, glial fibrillary acidic protein; NfL, neurofilament light chain.

In group 2, serum tau concentration was significantly different after day 7 blast exposure (z = −2.272, *p* = 0.023) (Figure 3A). The median concentration of tau at day 1 was 0.25 pg/mL (0.23–0.35) and day 7 blast exposure was 0.32 pg/mL (0.25–0.40). Serum p-tau181 concentration was significantly elevated after day 7 blast exposure (z = −2.158, *p* = 0.031) (Figure 3B). The median concentration of p-tau181 at baseline was 0.89 pg/mL (0.79 − 1.26) and 1.14 pg/mL (0.99 − 1.21) in day 7 blast exposure. Concentration of GFAP level was not significant after day 7 blast exposure (z = −0.057, *p* = 0.955) (Figure 3C). The levels of NfL was significantly different after day 7 blast exposure (z = −2.158, *p* = 0.031) (Figure 3D). The median concentration of NfL at day 1 was 5.96 pg/mL (4.43–6.89) and day 7 blast exposure was 6.13 pg/mL (5.34–7.11).

## Discussion

Our study investigated the impact of blast-related training on serum biomarkers in military personnel. These findings show similar trends in the reduction of serum GFAP levels after exposure to repetitive low-level blasts during the training program on day 2 (Boutté et al., 2019; Tschiffely et al., 2020). Here, we report that blast-related training results in acute changes in the axonal markers of tau, p-tau181, and NfL. Specifically, the concentration of tau and NfL levels were significantly elevated after day 7 blast exposure compared to pre-blast. Notably, serum p-tau181 level was significantly elevated at days 2 and 7 following blast exposure. Therefore, these proteins may serve as a potential candidate biomarker of blast-related injury in military training.

We observed an elevated level of tau in the acute and subacute timepoints following sports injury (McCrea et al., 2020; Giza et al., 2021). The concentration of tau and NfL has been shown higher in concussion and predicted symptom resolution following concussion (Shahim et al., 2018; Pattinson et al., 2020a). Proteins tau and NfL levels are more than acute markers of axonal injury but persistently elevated in chronic repetitive TBI, which is associated with neurobehavioral outcomes (Olivera et al., 2015; Pattinson et al., 2019). Further, we observed p-tau to be higher after blast, which is a novel finding. Tau phosphorylation plays an important role in the physiological function of tau protein regulation in the maintenance of the homeostasis of microtubules and the pathogenesis of neurodegenerative disorders (Rajmohan and Reddy, 2017; Katsumoto et al., 2019). In previous studies level of tau, p-tau181, and NfL proteins were significantly higher in chronic mTBI with persistent symptoms of post-traumatic stress disorder and depression (Kenney et al., 2018; Pattinson et al., 2019, 2020b). Recently, we have shown that extracellular vesicle tau and NfL proteins were associated with behavioral outcomes in military personnel (Edwards et al., 2021; Guedes et al., 2021). Elevation of p-tau181 was observed in many different brain regions and abnormal p-tau accumulation in astroglial, that may associate with behavioral changes following chronic repetitive blast exposure in preclinical model (Dickstein et al., 2021). Abnormal aggregation of p-tau causes synaptic impairment, neuronal dysfunction, and the formation of NFTs and is a key pathological feature of CTE (Rajmohan and Reddy, 2017; Katsumoto et al., 2019). Therefore, changes in these biomarker levels reflect axonal damage or regeneration that induced brain function impairment after repetitive low-level blast exposure. Additional studies of longitudinal change of these biomarkers are needed to determine the implication of these findings.

Our study has some limitations, including a small sample size and lack of gender and racial diversity, as well as lack of clinical symptoms over-time. Despite these limits, this study indicates that blast exposure results in changes in serum biomarkers of axonal injury. Notably, the level of p-tau181 protein was significantly elevated after blast exposures in both cohorts, which suggested that phosphorylated tau at threonine 181 may serve as an early biomarker of axonal injury in low-level blast exposures. However, the level of tau and NfL were not significant differences after immediate blast exposure, unlike the other cohort with 2 days intervals. We observed that 12 out of 19 participants were elevated in tau level and only 9 out of 19 participants were elevated in the NfL level in this group. Our plausible explanation is that some of these individuals may expose to low severity events of blast compared to the other participants. Previous studies showed that proteins biomarkers of GFAP and inflammatory cytokines were strongly associated with blast exposure levels (Gill et al., 2017; Tschiffely et al., 2020). We suggested that these serum biomarkers changes in our study may also impact by the level of blast exposure during training. However, we did not measure the blast exposure levels in this cohort. Additional larger studies with equal populations are needed to confirm these findings. In addition, a longitudinal study of these serum biomarkers changes is needed to evaluate the effect of blast exposure over time. These findings suggest that blast exposure is associated with acute changes of serum tau, p-tau181, and NfL level in military personnel. In conclusion, blast exposures in military personnel induced axonal damage which may serve as potential biomarkers of blast injury.

## Data Availability Statement

The raw data supporting the conclusions of this article will be made available by the authors, without undue reservation.

## Ethics Statement

The studies involving human participants were reviewed and approved by Institutional Review Board Committee at Washington University and U.S Army Fort Leonard Wood. The patients/participants provided their written informed consent to participate in this study.

## Author Contributions

All authors listed have made a substantial, direct, and intellectual contribution to the work, and approved it for publication.

## Author Disclaimer

The opinions or assertions contained herein are the private views of the authors, and are not to be construed as official, or as reflecting true views of the Uniformed Services University, Department of the Army, Department of the Navy, Department of Defense, or the U.S. Government.

## Conflict of Interest

The authors declare that the research was conducted in the absence of any commercial or financial relationships that could be construed as a potential conflict of interest.

## Publisher’s Note

All claims expressed in this article are solely those of the authors and do not necessarily represent those of their affiliated organizations, or those of the publisher, the editors and the reviewers. Any product that may be evaluated in this article, or claim that may be made by its manufacturer, is not guaranteed or endorsed by the publisher.
